# *RAB42* Promotes Glioma Pathogenesis *via* the VEGF Signaling Pathway

**DOI:** 10.3389/fonc.2021.657029

**Published:** 2021-11-29

**Authors:** Baoling Liu, Quanping Su, Bolian Xiao, Guodong Zheng, Lizhong Zhang, Jiawei Yin, Lijuan Wang, Fengyuan Che, Xueyuan Heng

**Affiliations:** ^1^ Central Laboratory, Key Laboratory of Tumor Biology, Key Laboratory of Neurophysiology, Linyi People’s Hospital, Linyi, China; ^2^ Department of Neurosurgery, Linyi People’s Hospital, Linyi, China; ^3^ Neuropathological laboratory, Linyi People’s Hospital, Linyi, China; ^4^ Department of Hematology, Linyi People’s Hospital, Linyi, China; ^5^ Department of Neurology, Linyi People’s Hospital, Linyi, China

**Keywords:** glioma, RAB42, vascular endothelial growth factor (VEGF), tumorigenesis, CGGA

## Abstract

Angiogenesis plays an important role in tumor initiation and progression of glioma. Seeking for biomarkers associated with angiogenesis is important in enhancing our understanding of glioma biologically and identifying its new drug targets. RNA-sequencing (RNA-seq) data and matched clinical data were downloaded from the CGGA database. A series of filtering analyses were performed to screen for reliable genes: survival, multivariate Cox, ROC curve filtration, and clinical correlation analyses. After immunohistochemical verification, *RAB42* was identified as a reliable gene for further single gene analysis. Afterwards, we performed gene set enrichment analysis (GSEA) and co-expression analysis to establish the related molecular mechanisms and signal pathways in glioma. Finally, the gene functions and the mechanisms were investigated *in vitro* experiments. A total of 23270 mRNA expression and 1018 glioma samples were included in this study. After the three filtering analyses, we selected ten genes for immunohistochemical verification: *KLHDC8A, IKIP, HIST1H2BK, HIST1H2BJ, GNG5, FAM114A1, TMEM71, RAB42, CCDC18*, and *GAS2L3*. Immunostaining demonstrated that RAB42 was significantly expressed on the membrane of glioma tissues but not in normal tissues. These results were verified and validated in GEPIA datasets, and the association between *RAB42* with clinical features was also evaluated. Analysis of gene functions indicated that *RAB42* activated VEGF signaling pathways and the mechanism was associated with natural killer cell mediated cytotoxicity, JAK-STAT signaling pathway and apoptosis pathways by PI3K/AKT in gliomas. Experiments *in vitro* suggested that the proliferation and invasion of glioma cells might be inhibited after downregulating of *RAB42*. And the tumorigenesis promotion of *RAB42* may relate to the activation of VEGF signaling pathway. Taken together, this study shows that the overexpression of *RAB42* is an independent prognostic factor of adverse prognosis. Its pro-oncogenic mechanism may be associated with the activation of VEGF signaling pathways.

## Introduction

Approximately 100,000 new cases of diffuse glioma are diagnosed globally every year (1, 2). According to morphological criteria of 2007 WHO guidelines for classifying gliomas, diffuse glioma is graded Stage II-IV. Glioblastoma (WHO IV, GBM) accounts for 70-75% of all diffuse gliomas. Despite the multiple treatment options including surgery, radiotherapy, and chemotherapy, the median overall survival of GBM is only 14-17 months (3).

Tumor angiogenesis is an important pathological process in the development of malignant tumors and refers to the formation of new blood vessels from pre-existing blood vessel networks (4). Glioblastoma is defined as lethal cancer by angiogenesis (5), which involves various mechanisms (6). Overlapping and intricate signaling pathways participate in the regulation of angiogenesis (7). As early as 25 years ago, the important role of vascular endothelial growth factor (VEGF) in vasculogenesis and angiogenesis had been identified, isolated, and cloned (8). Although VEGF has been demonstrated to be important in physiological vascular homeostasis, it contributes to the pathogenesis of malignant tumors by supporting growth and metastasis (9). The VEGF is overexpressed in the majority of human cancers and is correlated with vascular density, tumor invasiveness, metastasis, and prognosis (9). Several therapeutic approaches targeting the inhibition of the VEGF signaling pathway have been devised. Bevacizumab, a VEGF pathway inhibitor, has been approved for other malignancies in addition to metastatic colorectal cancer, including glioblastoma multiforme (8). However, Bevacizumab has displayed limited effectiveness in GBM as shown by relapse or progression of the tumor after 6 months of treatment (10). While numerous studies have increased the understanding of tumorigenesis and drug resistance, the mechanisms of glioma pathogenesis *via* the VEGF signaling pathway remain unclear.

With the development of high-throughput sequencing and bioinformatics, many tumor biomarkers of glioma, which is of great significance to the diagnosis and treatment of patients, have been identified (11). However, much more reliable biomarkers are needed in the diagnosis and decision-making processes. In this study, the filtering of the whole datasets of RNA-seq from the CGGA database allowed for the identification of 131 genes. Ten genes were screened for immunohistochemistry verification after bibliography data retrieval. The RAB42 was then screened for further identification. After a series of bioinformatics analysis and experiments *in vitro*, we found that RAB42 promotes glioma pathogenesis *via* the VEGF signaling pathway.

## Materials and Methods

### Acquisition of RNA-seq and Clinical Data

Both RNA-sequencing (RNA-seq) and clinical data were downloaded from the CGGA database (http://www.cgga.org.cn). The data included mRNA sequencing and corresponding clinical characteristics in glioma on 1,018 Chinese cohorts. There were two batches in all the RNA-seq data: mRNAseq 693 (batch 1) and mRNAseq 325 (batch 2). We performed batch correction and integration using the “sva” and “limma” package in R (version 4.0.2, https://www.r-project.org).

### Candidate Genes Filtering

A series of filtering analyses were performed to screen for reliable genes. First, we performed Kaplan–Meier (K-M) (12) and univariate Cox analyses with data from the whole RNA-seq datasets using “survival” and “survminer” packages in R. Genes with a KM and cox *P* values < 0.001, and five years differences > 0.2 were considered statistically significant. The above results obtained from the survival analysis were further analyzed by multivariate Cox analysis with a “survival” package in R. And the screening criterion was *p* Filter < 0.001. We then screened for genes with survival differences and independent prognostic value. To predict the accuracy, we further performed receiver-operating-characteristic (ROC) curves on the expression of these genes, by the “survivalROC” package in R by defining the filter criterion as the area under the curve (AUC) > 0.7. Finally, the expression results of the three filtering and clinical information were applied to perform clinical correlation analysis. And *P<0.05* was considered to be statistically significant.

### Immunohistochemistry

After filtering analyses, we screened reliable mRNA from RNA-seq data. To further verify the expression of these genes in clinical tissue specimens, ten genes (*KLHDC8A, IKIP, HIST1H2BK, HIST1H2BJ, GNG5, FAM114A1, TMEM71, RAB42, CCDC18*, and *GAS2L3*) were selected for re-verification. The histological samples of different grades of glioma patients in Linyi People’s hospital were reviewed and selected for formalin-fixed paraffin-embedded (FFPE) tissue material for immunohistochemistry (13). Brain tissues after trauma embedded in paraffin were chosen as normal control. We randomly selected 30 glioma cases (10 cases each for grades II, III, and IV) and 10 cases of brain trauma patients as normal controls for the protein expression re-verification of the 10 genes. All the tissues were cut into 5μm thick sections for immunohistochemistry staining. All the slides were incubated at 70°C for at least 30 mins. This was followed by deparaffinization, rehydration, boiling with EDTA antigen retrieval buffer, and then incubation with 10% goat serum for 1 h to block the nonspecific binding sites. After the incubation with the primary antibodies at 37°C for 1 hour (anti-KLHDC8A, 1:200 dilution, Abcam; anti-IKIP, 1:200 dilution, Abcam; anti-HIST1H2BK,1:200 dilution, Invitrogen; HIST1H2BJ,1:200 dilution, Invitrogen; anti-GNG5, 1:200 dilution, SIGMA; anti-FAM114A1, 1:200 dilution, proteintech; anti-TMEM71, 1:200 dilution, Abcam; anti-RAB42, 1:200 dilution, USBiological; CCDC18, 1:200 dilution, SIGMA and anti-GAS2L3, 1:200 dilution, Abcam), the sections were incubated with appropriate secondary antibodies at 37°C for 30 mins (1:1000, ZSGB-Bio, Beijing, China). The slides were exposed to HRP substrate 3,3-diaminobenzidine (DAB) (Tiangen, Beijing, China) and then dehydrated by dipping sequentially through 70%, 95%, 100% ethanol, and xylene before mounting (Neutral balsam; Solarbio).

### Single Gene Analysis

The reliable gene, *RAB42*, was identified for further analysis. *RAB42* gene expression data and matched clinical information were used to perform survival, univariate, multivariate, and plot ROC curves analyses using “survival”, “survminer”, and “survivalROC” packages in R. The correlation between *RAB42* gene expression and clinical traits was then validated. The “beeswarm” packages in R was used for graphic visualization. Further re-verification of *RAB42* expression and survival analysis was performed in the Gene Expression Profiling Interactive Analysis (GEPIA; http://gepia2.cancer-pku.cn) and the Cancer Genome Atlas (TCGA) database. GEPIA is an interactive web server containing RNA sequencing data and clinical data based on 9,736 tumor and 8,587 normal samples from the TCGA and GTEx databases (14). In addition, we downloaded transcriptome expression profiles and clinical data from the TCGA database (https://portal.gdc.cancer.gov). Survival analysis was performed with the “survival” package in R(version 4.0.2, https://www.r-project.org).

### Gene Set Enrichment Analysis

Samples were divided into high- and low-risk population phenotypes based on *RAB42* gene expression levels. Gene set enrichment analysis (GSEA) (https://www.gsea-msigdb.org/gsea/index.jsp) was then used to assess related molecular mechanisms and signal pathways in glioma patients (15). Gene sets with a nominal P < 0.05 and a false discovery rate (FDR) < 0.25 were considered statistically significant.

### Co-Expression Analysis

To further screen for the genes co-expressed with *RAB42*, we performed co-expression analysis using the “limma” package in R. Genes with p<0.001, and correlation coefficient >0.5 were considered as co-expressed genes. The gene sets of interest were previously identified through GSEA analysis. The overlapping genes between the co-expressed genes with *RAB42* and genes in the designated Gene sets were chosen as function-related genes. Venn diagram was plotted using the “VennDiagram” package in R. Furthermore, the top 20 genes positively and negatively associated with *RAB42* were exhibited in the heatmap using the “ pheatmap” package in R. The top 5 genes positively and negatively associated with *RAB42* were used to generate a circular plot by “Corrplot” and “Circlize” packages in R.

### Cell Culture, Transfection, and Generation of Stable Cell Lines

All the glioblastoma cell lines SNB-19, KNS-89, CRT, U87, U251, A172, TG905 and SF295 were kindly provided by Central Laboratory of Linyi People’s Hospital. All the cell lines were free from mycoplasma contamination with a certificate of authentication with STR profiling. They were cultured in Dulbecco’s modified Eagle’s medium (DMEM, Gibco, Thermo Fisher Scientific, Shanghai, China), 10% FBS (Thermo Fisher Scientific), penicillin (100 U/mL), and streptomycin (100 µg/mL) at 37°C, and 5% CO2 air. Cells were digested using Trypsin‐EDTA (0.05%)(Beijing Soledad Bao Technology Co, Beijing, China) for cell passages.

The *RAB42*-knockdown stable cell lines (SNB19-sh-*RAB42*) were obtained by transfecting pSilencer 4.1-CMV-puro-RAB42 small interfering RNA (siRNA) vector into SNB-19 cells and selecting for stable clones in the presence of 0.5 mg/mL puromycin. And empty vectors were transfected as a control (SNB19-sh-NC).

### Cell Proliferation Assay

Cell proliferation was tested using CCK8 cell proliferation assay kit (Beyotime Biotechnology, Shanghai, China). First, we plated cells at a density of 5,000 cells/well in 96‐well plates. Then, cells were allowed to attach 4 h, after which cells were collected at 0h, 1d, 2d, 3d, 4d and 5d. After adding 10 μl of CCK‐8 solution per well and incubating for 1 to 4h, we measured the absorbance at 450 nm using an enzyme calibrator (MolecularDevices, Sunnyvale, CA). The formula for calculating the cell viability was as follows: (mean OD treated wells)/(mean OD control wells) × 100%.

### Transwell Assays

Transwell migration and transwell invasion assay were performed to analyze migration and invasion ability using transwell chambers (Corning Inc., Corning, USA). Cells were planted into the upper chamber in the serum‐free culture medium with the density of 1×10^5^ cells/well. The lower chambers was added with 800 μl medium containing 20% FBS. After incubating for 24 h, cells were fixed with 4% PFA and stained with crystal. After wiping cells in the upper chamber with a cotton swab, the cells were observed under an inverted microscope and photographed. Cell invasion assays were performed in chambers precoated with matrigel (Corning Inc., Corning, USA) with the cells seeding density of 10×10^5^ cells/well.

### Colony Formation Assay

Cells were digested, resuspended and eeded onto six-well plates at the density of 200 cells per well. After 2 weeks of culturing in an incubator with 5% CO2 at 37°C, cells were fixed and stained using crystal violet. Then, colonies that contained more than 50 cells were counted and calculated.

### Real-Time Quantitative PCR and PCR Microarray

RT-PCR was conducted to determine the gene expression of *RAB42* and endogenous pluripotency genes. First, total RNA of cells was extracted by RNA extraction kit (GeneCopoeia, Rockville, MD, USA) following manufacturer’s instructions. Then, the concentration of the RNA was detected for further study. Next, cDNA was synthesized using a first-strand cDNA synthesis kit (GeneCopoeia, Rockville, MD, USA). Primers used for amplification of *RAB42* gene and the reference gene, *GAPDH*, are described in **Supplementary Table S5**. Relative quantification of the gene expression level was calculated by 2^−(ΔΔCt)^ method (16). The qRT-PCR analysis was repeated in three biological and three technical replications.

VEGFR PCR Array (WCgene, Biotechnology, Shanghai China) was utilized to the investigation of further molecular mechanism. The specific gene primers were designed by Wcgene Biotech (WCgene, Shanghai, China). RNA extraction and cDNA synthesis were performed as described. PCR reactions were performed using real-time 7500 PCR system (Applied Biosystems).And the PCR reaction procedure was as follows: 95°C For 10 min, then 40 cycles at 95°C for 15 sec and finally at 60°C for 1 min. The results were analyzed as previously described.

### Western Blot Analysis

Western blot (WB) analysis using for detecting protein expressions was performed following standard techniques. Total protein was extracted using protein extraction kit (Solibao Technology Co. Ltd, Beijing, China). The protein concentration standard curve was plotted and then the protein concentrations were determined using BCA protein concentration assay kit (Biosharp, Hefei, China). After SDS-PAGE electrophoresis and wet transfer, the target protein was transferred to PDVF membrane (Solarbio, Beijing, China). Following blocking with 5% nonfat dry milk, the primary and secondary antibodies were incubated for 2 and 1 hour respectively at room temperature. Membranes were then washed and developed by ECL (Bio-Rad, 1705061).

### Statistical Analysis

All data of bioinformatics analysis were conducted by R language (version 4.0.1). Kaplan–Meier (K-M) and univariate Cox analyses was used to compare the survival differences between groups using “survival” and “survminer” packages in R. Genes with a KM and cox P values < 0.001, and five years differences > 0.2 were considered statistically significant. Spearman’s correlation was used to screen for the genes co-expressed with *RAB42* by “limma” package in R. Genes with p<0.001, and correlation coefficient >0.5 were considered as co-expressed genes. All data of experiments *in vitro* are expressed as mean ± standard deviation (unless otherwise shown). Student’s t‐tests were used to determine the significance of differences between two groups, and one‐way ANOVA and Tukey’s *post hoc* test were used to determine differences among multiple groups. P<0.05 was considered to indicate a statistically significant difference.

## Results

**Figure 1:**
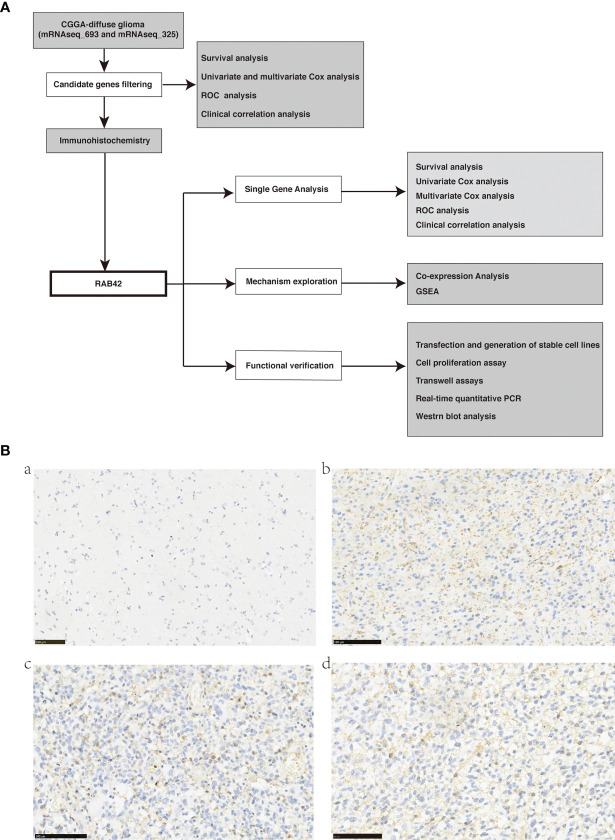
**(A)** Flow chart of data download, filtering, validation, and mechanism exploration; **(B)** Representative images of RAB42 in primary glioma patient tissues (Tumors were graded according to the WHO classification) and control normal tissues by immunohistochemical staining (magnification, 200×). (a) RAB42 expression was not visible in control normal tissues. (b) RAB42 was found clearly expressed on the membrane of glioma tissues(WHO, Grade II); (c) RAB42 was found strongly expressed on the membrane of glioma tissues(WHO, Grade III); (d) RAB42 was found strongly expressed on the membrane of glioma tissues(WHO, Grade IV).

The procedure of this study was presented in the flow chart (**Figure 1A**).

### Data Collection and Candidate Genes Filtering

A total of 23270 mRNA expression and 1018 glioma samples were included for subsequent analysis. After performing survival analysis, 5112 genes showing survival differences were obtained (**Supplementary Table 1**). By screening for the genes that could be used as independent prognostic factors using Multivariate Cox analysis, a total of 2630 genes were obtained (**Supplementary Table 2**). From the ROC curve filtration, using AUC>0.7 as the screening criterion, 131 reliable genes were identified to have good performance of survival prediction (**Supplementary Table 3**). The relationship between the 131 genes and clinical features, including Primary/Recurrent/Secondary type, LGG/GBM histology, WHO grade, Age, Grade, Radiotherapy status, Chemotherapy status, IDH mutation status, and 1p19q codeletion status, have been shown in **Supplementary Table 4**. Ten among these 131 genes (*KLHDC8A, IKIP, HIST1H2BK, HIST1H2BJ, GNG5, FAM114A1, TMEM71, RAB42, CCDC18*, and *GAS2L3*) were selected based on Bibliography Retrieval for subsequent immunohistochemical verification.

### Immunohistochemistry Verification: RAB42 Was Upregulated in Glioma but Not Expressed in the Normal Brain Tissue

We performed immunohistochemistry staining for the ten selected genes on glioma and normal brain tissues. Eight of them showed no expression in tumor tissues. HIST1H2BK was strongly expressed in the nucleus of both glioma and normal brain tissues. RAB42 was markedly expressed on the membrane 429 of glioma tissues (**Figures 1B–D**, brown) particularly in 430 glioblastoma (Grade IV), and not on the normal brain tissues 431 (**Figure 1A**). Thus, we validated that RAB42 is differentially 432 expressed between tumor and normal tissues using 433 434 immunohistochemistry staining.

**Figure 2:**
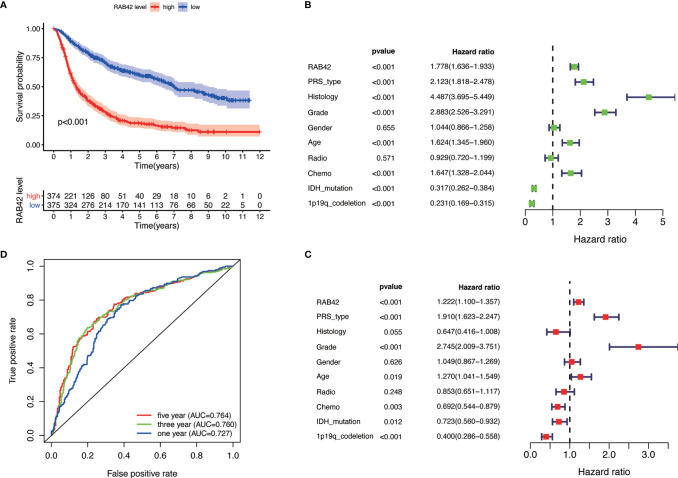
Single gene analysis of *RAB42* using the CGGA database (LGG+GBM, n = 749). **(A)** Survival curve of glioma patients in high- and low- *RAB42* groups. **(B)** Forest plot of univariate analysis. *RAB42* (HR = 1.778; 95% CI = 1.36–1.933; P < 0.001), age, PRS type, histology, grade and chemo were independent poor prognostic factors, while IDH mutation and 1p19q codeletion were favorable prognostic factor. **(C)** Forest plot of multivariate analysis showing *RAB42* as an independent prognostic factor (HR = 1.222; 95% CI= 1.100–1.357; P < 0.001). **(D)** ROC analysis of *RAB42*. AUC, the area under the curve. The area under the ROC curve (AUC) of 1-year, 3-year, and 5-year overall survival was 0.764, 0.760, and 0.727 respectively.

### Bioinformatics Analysis Revealed the Pro-Carcinogenic Effect of *RAB42*

Based on our findings, we analyzed the tumor-promoting effect of *RAB42* using bioinformatics. Survival analysis indicated that low *RAB42* expression in CGGA glioma (including GBM and LGG) tissues predicted favorable survival outcome and vice versa (Kaplan-Meier survival analysis; P < 0.001; **Figure 2A**). As shown in results of the univariate Cox analysis, *RAB42* (HR = 1.778; 95% CI = 1.36–1.933; P < 0.001), age, PRS type, histology, grade, and chemotherapy status were independent poor prognostic factors, while IDH mutation and 1p19q codeletion were well described prognostic factor (**Figure 2B**). *RAB42* was still identified as an independent prognostic factor in a multivariable analysis (HR = 1.222; 95% CI= 1.100–1.357; P < 0.001), implying that it could be a powerful indicator of prognosis in glioma portending poor prognosis (**Figure 2C**). To depict the accuracy of RAB42 for predicting prognostic in glioma patients, we plotted ROC curves using the data downloaded from the CGGA database. The area under the ROC curve (AUC) is a measure of the accuracy of the marker prediction, where AUC = 0.50 means no discrimination, and AUC = 1.0 means perfect prediction (17). The ROC curve analysis indicated that RAB42 had high diagnostic accuracy, and the area under the ROC curve (AUC) of 1-year, 3-year, and 5-year overall survival was 0.764, 0.760, and 0.727, respectively (**Figure 2D**).

**Figure 3:**
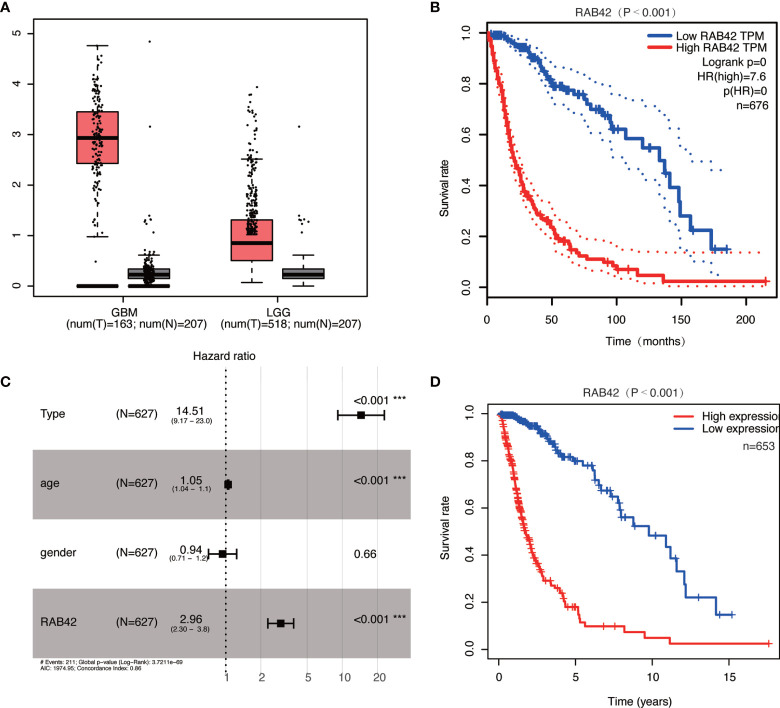
Relationship between *RAB42* expression and prognosis of glioma patients based in the GEPIA and TCGA database. **(A)** *RAB42* was significantly upregulated both in GBM and LGG in the GEPIA database. **(B)** Survival curve of *RAB42* expression levels and overall survival in the GEPIA database (P < 0.001). **(C)** Forest plot of multivariate analysis showing *RAB42* as an independent prognostic factor (HR = 2.96; 95% CI = 2.30–3.80; P < 0.001) in TCGA database. **(D)** Survival curve of GBM and LGG patients in high- and low- *RAB42* groups in TCGA database *** means P < 0.001.

In GEPIA datasets, we found that the expressions of *RAB42* in GBM and LGG were upregulated (**Figure 3A**). Survival curve indicated that the high expression of the *RAB42* was associated with poor prognosis (**Figure 3B**). After collecting transcriptome expression profiles and clinical data, 695 samples were downloaded from the TCGA database, including 167 GBM tissues and 528 LGG samples. Multivariate Cox regression analysis showed *RAB42* (HR = 2.96; 95% CI = 2.30–3.80; P < 0.001), PRS type (HR = 14.51; 95% CI = 9.17–23.00; P < 0.001) and age (HR = 1.05; 95% CI = 1.04–1.1; P < 0.001) were independent poor prognostic factors. This is consistent with CGGA results indicating that *RAB42* could be a poor prognostic predictor in glioma (**Figure 3C**). Additionally, overall survival analysis of *RAB42* expression in GBM and LGG revealed that patients with high expression of *RAB42* had a significantly worse prognosis (**Figure 3D**). This is consistent with our previous analysis in CGGA database.

**Figure 4:**
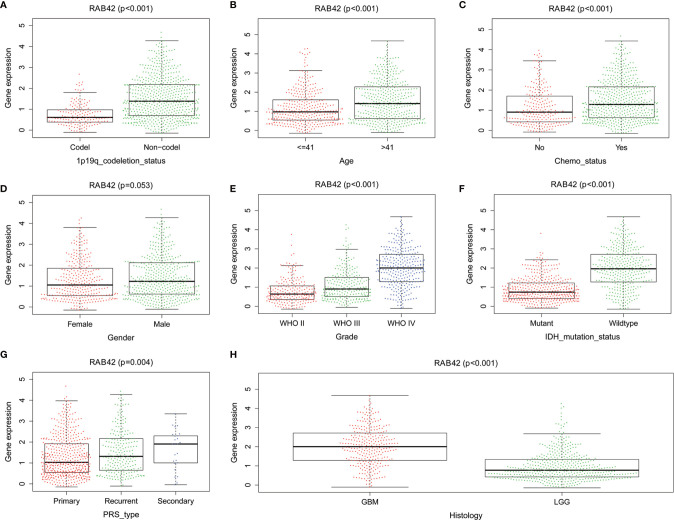
The relationship between *RAB42* expression and clinical traits using the CGGA database. Differential expression of *RAB42* was significantly related to **(A)** 1p19q codeletion status (Codel, n = 155; Non-codel, n = 594), **(B)** age (≤41, n = 342; ≥41, n = 407), **(C)** chemotherapy status (No, n = 229; Yes, n = 520), **(D)** Gender (Female=322, Male=427), **(E)** Grade (WHO II, n = 218; WHO III, n = 240;WHO IV, n = 291), **(F)** IDH mutation status (Mutant, n = 410; Wildtype, n = 339), **(G)** PRS type (Primary, n = 502; Recurrent, n = 222; Secondary, n = 25) and **(H)** Histology (GBM=288;LGG=461).

To establish the relationship between *RAB42* expression and clinical traits, we analyzed their relevance using the 1,018 samples from the CGGA database. Results showed that the expression of *RAB42* was closely related to 1p19q codeletion status, age, chemotherapy status, grade, IDH mutation status, PRS type, and histology (**Figure 4**).

### Gene Set Enrichment Analysis of *RAB42*

**Figure 5:**
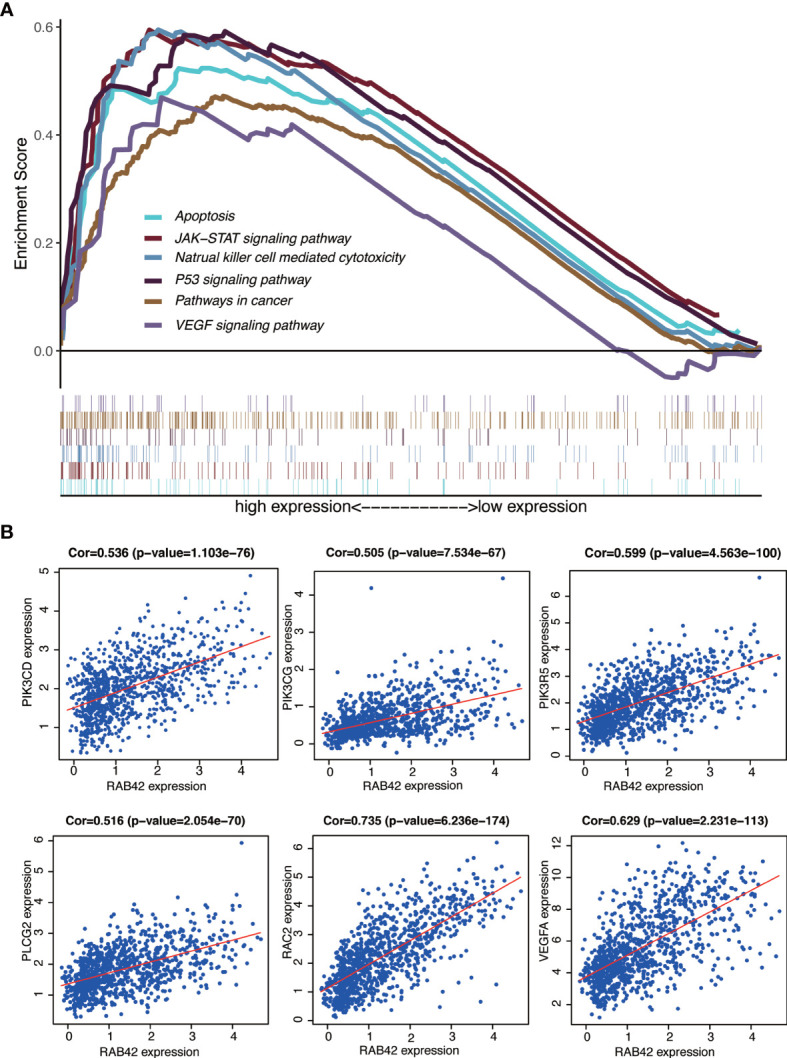
GSEA of *RAB42* and identification of go-expression genes. **(A)** GSEA enrichment analysis of *RAB42* (GBM LGG, n = 749). *RAB42* positively correlates with apoptosis, natural killer cell mediated cytotoxicity, P53 signaling pathway, pathways in cancer, JAK-STAT signaling pathway and VEGF signaling pathway.**(B)** Correlation curves of *RAB42* and *PIK3CD, PIK3CG, PIK3R5, PLCG2, RAC2*, and *VEGFA*.

The signaling pathways enrichment in high and low *RAB42* expression groups were analyzed using GSEA software. MSigDB C2 collection (c2.cp.kegg.v7.1.symnols.gmt) was chosen and gene sets with significant differences (FDR < 0.05) downloaded. It turned out that 170 signaling pathways were enriched in the high-*RAB42* expression group. Of these, 151 gene sets were upregulated and multiple signaling pathways seemed to be very closely related to cancer. Six pathways showing significant enrichment associated with cancer are shown in **Figure 5A**. *RAB42* was positively correlated with apoptosis, natural killer cell mediated cytotoxicity, P53 signaling pathway, pathways in cancer, JAK-STAT signaling pathway and VEGF signaling pathway.

### Identification of Co-Expression Genes

Gene co-expression analysis with the full expression profile identified 1280 relevant genes that were closely correlated with *RAB42*. Heat maps of the top 20 negatively and positively correlated genes are shown in **Supplementary Figure 1A** and a circular plot of the top negatively and positively related genes shown in **Supplementary Figure 1B**. We performed a series of alignments with related genes of *RAB42* and genes in each pathway to characterize downstream mechanisms and explored key genes. The intersection between the 1280 related genes and genes of pathways in cancer and VEGF signaling pathways highlighted six genes: *PIK3CD, PIK3CG, PIK3R5, PLCG2, RAC2*, and *VEGFA*. Further analyses on the relationship between these genes and apoptosis, natural killer cell mediated cytotoxicity and JAK-STAT signaling pathways showed that *PIK3CD, PIK3CG*, and *PIK3R5* were common key genes (**Supplementary Figure 2**). These results indicated that *RAB42* activated VEGF signaling pathways and was associated with apoptosis, natural killer cell mediated cytotoxicity and JAK-STAT signaling pathways by PIK3 signaling pathway in gliomas. Finally, we plotted the correlation curves of *RAB42* and *PIK3CD, PIK3CG, PIK3R5, PLCG2, RAC2*, and *VEGFA* (**Figure 5B**). The expression of all the six genes were correlated positively with *RAB42* expression (*PIK3CD*, *P*=1.103e-76; *PIK3CG*, *P*=7.534e-67; *PIK3R5*, *P*=4.563e-100; *PLCG2*, *P*=2.054e-70; *RAC2*, *P*=6.236e-174, and *VEGFA*, *P*=2.231e-113).

### Validation of *RAB42* Gene Functions *In Vitro*

**Figure 6:**
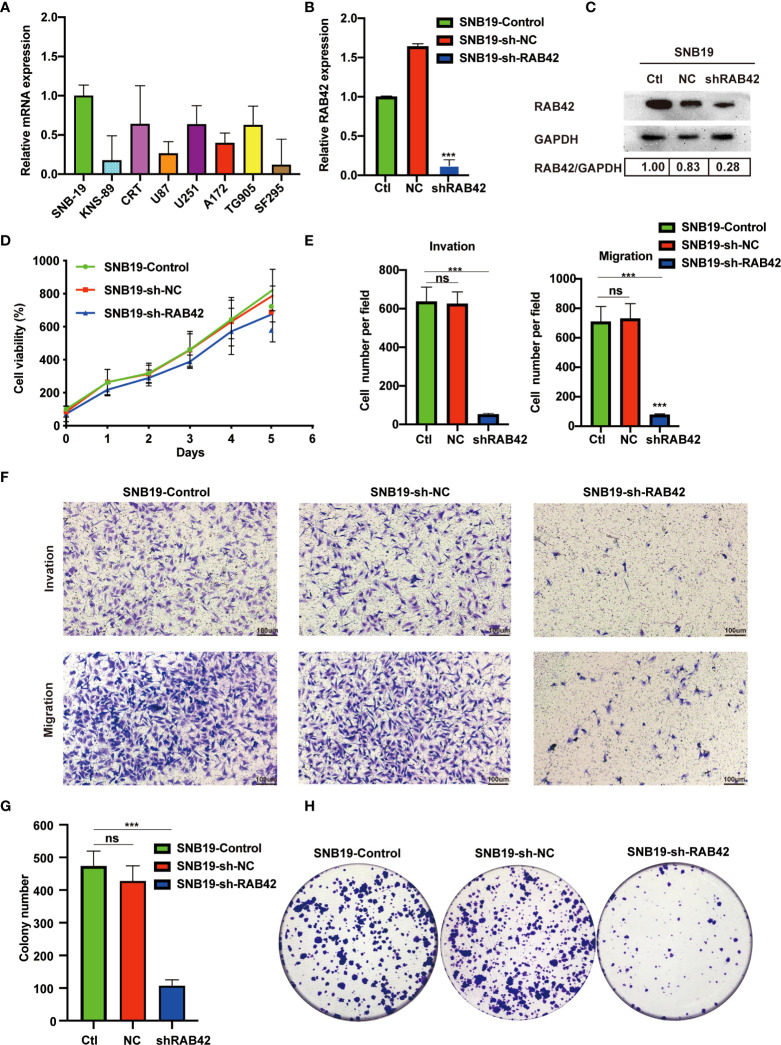
Validation of *RAB42* gene functions *in vitro*. **(A)** Relative mRNA expression of SNB-19, KNS-89, CRT, U87, U251, A172, TG905 and SF295. **(B)** Relative expression of *RAB42* in SNB19 cell lines after *RAB42* knockdown (SNB19-sh-*RAB42*), parental cell line SNB19 (SNB19-Control) and control corresponding to the parental cell line transfected with the empty expression vector (SNB19-sh-NC). **(C)** Relative protein expression of SNB19-sh-*RAB42*, SNB19-Control and SNB19-sh-NC. **(D)** Cellular viability by CCK8 assay. **(E)** Quantification of invasion and migration assays. **(F)** Transwell invasion and migration assays. **(G)** Quantification of colony number in clonogenic assay. **(H)** Clonogenic assays. “ns” means “not significant”, *** means P < 0.001.

At the outset, we first detected the expression of *RAB42* by RT-PCR in eight cell lines (SNB-19, KNS-89, CRT, U87, U251, A172, TG905 and SF295). As showcased in **Figure 6A**, the SNB-19 cells, which exhibiting the highest expression level of *RAB42* among all the 8 cell lines, was selected as the targeted cell for further study. Efficient *RAB42* knockdown had been confirmed by PCR and WB (**Figures 6B, C**). Results of CCK8 assays showed downregulation of *RAB42* reduced cell proliferation of glioma cells (**Figure 6D**). As shown in transwell results (**Figures 6E, F**), *RAB42* knockdown inhibited invasion and migration of glioma cells. Clonogenic assay of cells showed similar results in **Figures 6G, H**. The above results suggested that the proliferation and invasion of glioma cells might be inhibited after downregulating of *RAB42*, which also suggesting the tumor‐promoting role of *RAB42* in glioma.

### Mechanism Verification by Which *RAB42* Promotes Glioma pathogenesis *via* the VEGF Signaling Pathway

**Figure 7:**
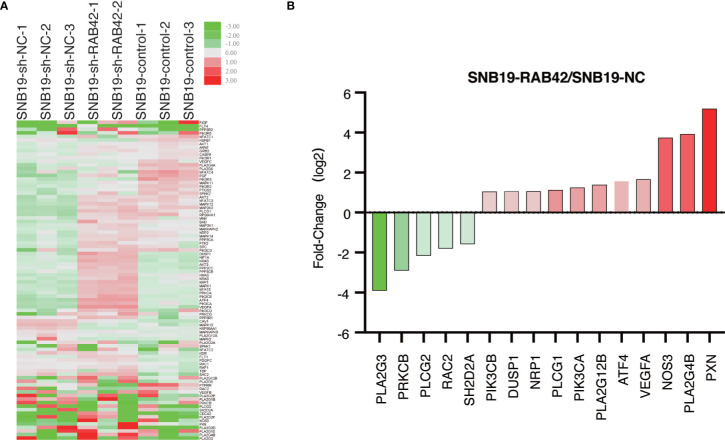
PCR microarray **(A)** Heat map depicting genetic alterations in VEGF signaling pathway after *RAB42* knockdown in glioma. **(B)** Selection of genes that were altered the most in SNB19-*RAB42* cells.

The stable *RAB42* knockdown cells (SNB19-sh-*RAB42*), parental cell line SNB19 (SNB19-Control) and control corresponding to the parental cell line transfected with the empty expression vector (SNB19-sh-NC) were sent for sequencing. As shown in the **Supplementary Table 6** and **Supplementary Figure 3**, after knocking down *RAB42*, the expression of *VEGFD, PIK3CD, PIK3R3* and *PIK3R1* changes due to the knockdown of *RAB42* in SNB19 cells. Meanwhile, a PCR microarray was performed to investigate genetic alterations in VEGF signaling pathway after *RAB42* knockdown in glioma. As shown in **Figure 7A**, 36 genes associated with VEGF signaling pathway were altered in SNB19-RAB42 cells. Among them, five genes (*PLA2G3, PRKCB, PLCG2, RAC2* and *SH2D2A*) that showed positive correlation between *RAB42*, while eleven genes (*PIK3CB, DUSP1, NRP1, PLCG1, PIK3CA, PLA2G12B, ATF4, VEGFA, NOS3, PLA2G4B* and *PXN*) showed negative correlation (**Figure 7B**) with significantly statistical difference. The results confirmed that the tumorigenesis promotion of *RAB42* may relate to the activation of VEGF signaling pathway. This is consistent with the former analyses.

## Discussion

Diffuse gliomas account for approximately 80% of malignant brain tumors (18, 19). Adult diffuse gliomas mainly include oligodendroglioma, oligoastrocytoma, astrocytoma, and glioblastoma according to histological features (20). In recent years, studies have shown how classical histopathology has failed to meet current clinical demand as compared with molecular reclassification (21, 22). These have resulted in a major revision of glioma classification in the 2016 WHO guideline by incorporating isocitrate dehydrogenase(IDH) status and 1p/19q codeletion diagnostics (23). Moreover, growing studies have demonstrated that a few functional molecules could be used as potential diagnostic and prognostic biomarkers in gliomas (24, 25). Therefore, the search for prognostic biomarkers that can improve the outcome in glioma is valuable for increasing our understanding and exploring therapeutic approaches to glioma.

The characterization efforts of the gene (for example, TCGA) have greatly enhanced our understanding of the biological process for glioma progression (26). The Chinese Glioma Genome Atlas (CGGA), is a web application for data storage and analysis for exploring brain tumors datasets over 2,000 samples from Chinese cohorts. More than 200 SCI papers have been published and more than 20 projects have been conducted using this database directly or indirectly. From the analysis of the gene expression data and clinical information of CGGA, Liu et al. found that *HIST1H2BK* could be a biomarker for LGG with poor prognosis (27). In this study, RNA-seq data of glioma and matched clinical data were downloaded from the CGGA database. After filtering by survival analysis, multivariate Cox, ROC curve, and clinical relevance, 131 genes were identified. All of the 131 genes significantly correlated with survival, and served as reliable independent predictors with high clinical relevance. Ten genes (*KLHDC8A, IKIP, HIST1H2BK, HIST1H2BJ, GNG5, FAM114A1, TMEM71, RAB42, CCDC18*, and *GAS2L3*) were then selected for immunohistochemical verification. As a result, *KLHDC8A, IKIP, HIST1H2BJ, GNG5, FAM114A1, TMEM71, CCDC18*, and *GAS2L3* were not expressed both in glioma and normal tissues. HIST1H2BK was strongly expressed in the nucleus of both glioma and normal tissue cells. Contrary, RAB42 was significantly expressed on the membrane of glioma tissues but not in normal tissues.

RAB42 is a member of the mammalian Rab family small GTPases (28). Consistent with our findings, a previous study showed that *RAB42* is one of the Seven genes used for the prognostic prediction in patients with glioma (29). This previous study mainly focused on the building of the seven genes prognostic model with minimum attention paid to *RAB42*. This study performed a single gene analysis of *RAB42* and found that it had as a tumor-promoting role in glioma and could be a powerful indicator of prognosis in glioma portending poor prognosis. Besides, a strong association was found between the expression of *RAB42* and multiple clinical features. To explore the possible mechanism of *RAB42* cancer-promoting functions, we further performed GSEA and co-expression analysis. The results indicated that *RAB42* activated VEGF signaling pathway and was associated with apoptosis, natural killer cell mediated cytotoxicity pathways and JAK-STAT signaling pathways by PI3K/AKT in gliomas.

Central malignant processes for glioma, neo-angiogenesis, and vascularity are important factors for glioma histopathological grading (30, 31). As an angiogenic growth factor produced by tumors, VEGF can often stimulate tumor angiogenesis (32). It has also been identified that VEGF plays a critical regulatory role in the angiogenic response of glioblastomas (33). Antiangiogenic therapy causes devascularization that inhibits tumor growth and VEGF is considered a functionally important drug target (34). Researchers have reported that PI3K/AKT pathway is involved in angiogenesis and that the suppression of the PI3K/AKT signal axis could inhibit new blood vessel formation by downregulating VEGF expression (35). Similar to previous studies, our findings indicated that *RAB42* functioned as an oncogene in glioma, activated VEGF signaling pathways, and regulated PI3K/AKT signal axis. Furthermore, our study found that potential mechanisms of *RAB42* were associated with apoptosis, natural killer cell mediated cytotoxicity pathways and JAK/STAT signaling pathway through the regulation of PI3K/AKT. The pleiotropic cascade mediated by JAK/STAT pathway is the principal mechanism for signal transduction of multiple cytokines and growth factors. It has been reported that the activation of the JAK/STAT pathway causes the inhibition of apoptosis and tumor initiation (36). Growing evidence suggests that angiogenesis and tumor progression are related to members of the innate immune system (37). As a part of the innate immunity, natural killer cell-mediated cytotoxicity is strongly associated with tumorigenesis and tumor development (38). In the present study, *RAB42* showed cancer-promoting functions by regulating multiple tumor-related signaling pathways, which is consistent with previous findings.

We found that the pro-oncogenic mechanism of *RAB42* could be through the activation of VEGF signaling pathways, which is novel and has potential clinical utility. In this study, *RAB42* was selected from a large number of candidate genes as the target gene for further investigation. A large amount of bioinformatic analysis demonstrated that the tumorigenesis promotion of *RAB42* may relate to the activation of VEGF signaling pathway. Subsequently, we experimentally validated that *RAB42* promoted the proliferation, migration and invasion of glioma and the pro-oncogenic mechanism of *RAB42* is associated with the activation of VEGF signaling pathways. However, this study had some limitations. We collected the RNA-seq data and clinical information from the CGGA dataset instead of getting our sequencing data from patients of our hospital. Besides, the sample size used for immunohistochemistry verification was not large enough and the information about clinical characters and survival data was not enough. In addition, the study mainly focused on bioinformatic analysis with limited experimental validation of the function and the specific mechanisms of action of *RAB42*. Therefore, more in-depth studies are needed to investigate the function and pro-oncogenic mechanism of *RAB42*.

## Conclusion

In conclusion, we explored the biological function of *RAB42* and mechanism in glioma. We used filtering analyses to screen for the reliable genes and performed immunohistochemical verification to identify the key gene, *RAB42*. Single gene analysis of *RAB42* showed that *RAB42* is an independent prognostic factor of adverse prognostic. *RAB42* expression is associated with multiple clinical features. Furthermore, functional *in vitro* experiments indicated that *RAB42* promoted the proliferation, migration and invasion of glioma and the pro-oncogenic mechanism of *RAB42* is associated with the activation of VEGF signaling pathways.

## Data Availability Statement

The datasets presented in this study can be found in online repositories. The names of the repository/repositories and accession number(s) can be found in the article/**Supplementary Material**.

## Ethics Statement

The studies involving human participants were reviewed and approved by the Human Research Ethic Committee of Linyi People’s hospital. The patients/participants provided their written informed consent to participate in this study.

## Author Contributions

BL conducted the experiments, wrote, and edited the manuscript. QS and BX provided experimental support. GZ provided glioma and traumatic brain tissue. LZ provided support during the experiment. JY reviewed and edited the manuscript. LW and FC contributed to analyze the data and draft the manuscript. XH contributed to the conception, design, and analysis of the manuscript. All authors contributed to the article and approved the submitted version.

## Funding

This research is supported by the postdoctoral innovation project of Shandong Province in 2020 (No.20203068), Medical and Health Technology Development Program in Shandong Province (No. 2017WS499), Medical Health Science and Technology Development Plan of Shandong Province (No.2019WS133) and Technology Development Plan Program of Linyi (No.202020001).

## Conflict of Interest

The authors declare that the research was conducted in the absence of any commercial or financial relationships that could be construed as a potential conflict of interest.

## Publisher’s Note

All claims expressed in this article are solely those of the authors and do not necessarily represent those of their affiliated organizations, or those of the publisher, the editors and the reviewers. Any product that may be evaluated in this article, or claim that may be made by its manufacturer, is not guaranteed or endorsed by the publisher.

## Supplementary Material

Supplementary Table 1 Results of survival analysis filtering.

Supplementary Table 2 Results of independent prognostic analysis filtering.

Supplementary Table 3 Results of ROC curve filtering.

Supplementary Table 4 Results of clinical relevance analysis filtering.

Supplementary Table 5 Primers used in the experiments

Supplementary Table 6 The differential gene expression of SNB19-WT vs SNB19-RAB42

Supplementary Figure 1 Identification of RAB 42 Co-expression Genes. **(A)** Heatmap of the top twenty genes positively and negatively correlated to *RAB42*. **(B)** A circular plot of the top five genes positively and negatively associated with *RAB42*. Red indicates a positive correlation whereas green indicates a negative correlation.

Supplementary Figure 2 The Venn diagrams showed the common key genes of *RAB42* related genes and genes in pathways in cancer, VEGF signaling pathway, apoptosis, natural killer cell mediated cytotoxicity and JAK-STAT signaling pathways.

Supplementary Figure 3 Heatmap and Volcano Plot of the differential gene expression.

## Abbreviations

GBM, Glioblastoma; VEGF, vascular endothelial growth factor; RNA-seq, mRNA sequencing; CGGA, Chinese Glioma Genome Atlas; ROC, receiver-operating-characteristic; AUC, area under curve; FFPE, formalin-fixed paraffin-embedded; GSEA, Gene Set Enrichment Analysis; IHC, immunohistochemistry; GEPIA, Gene Expression Profiling Interactive Analysis; TCGA, Cancer Genome Atlas; RT-PCR, Real-time quantitative PCR; WB, Western blot.
