# Corrigendum: RAB42 promotes glioma pathogenesis *via* the VEGF signaling pathway

**DOI:** 10.3389/fonc.2022.1034167

**Published:** 2022-10-07

**Authors:** Baoling Liu, Quanping Su, Bolian Xiao, Guodong Zheng, Lizhong Zhang, Jiawei Yin, Lijuan Wang, Fengyuan Che, Xueyuan Heng

**Affiliations:** ^1^ Central Laboratory, Key Laboratory of Tumor Biology, Key Laboratory of Neurophysiology, Linyi People’s Hospital, Linyi, China; ^2^ Department of Neurosurgery, Linyi People’s Hospital, Linyi, China; ^3^ Neuropathological laboratory, Linyi People’s Hospital, Linyi, China; ^4^ Department of Hematology, Linyi People’s Hospital, Linyi, China; ^5^ Department of Neurology, Linyi People’s Hospital, Linyi, China

**Keywords:** glioma, RAB42, vascular endothelial growth factor (VEGF), tumorigenesis, CGGA

In the published article, there was an error in **Figure 6**. In **Figure 6H**, the author mistakenly uploaded the “Control” image as the “NC” image. The corrected **Figure 6H** appears below.

**Figure 6:**
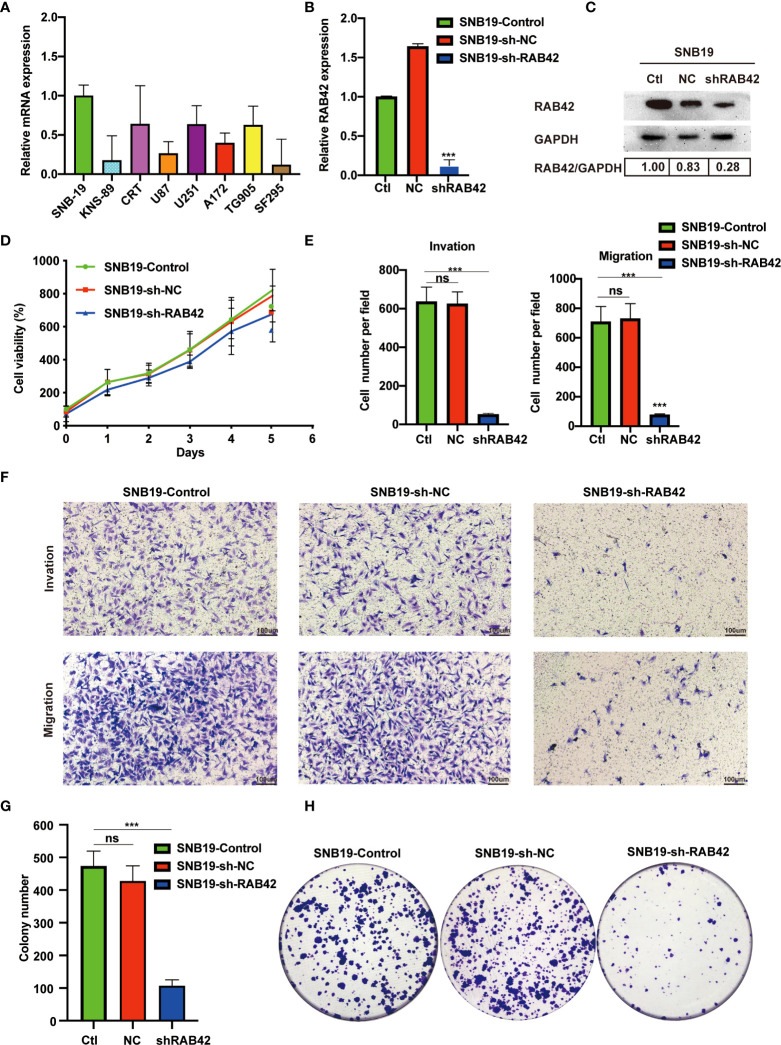
Validation of *RAB42* gene functions *in vitro*. **(A)** Relative mRNA expression of SNB-19, KNS-89, CRT, U87, U251, A172, TG905 and SF295. **(B)** Relative expression of *RAB42* in SNB19 cell lines after *RAB42* knockdown (SNB19-sh-*RAB42*), parental cell line SNB19 (SNB19-Control) and control corresponding to the parental cell line transfected with the empty expression vector (SNB19-sh-NC). **(C)** Relative protein expression of SNB19-sh-*RAB42*, SNB19-Control and SNB19-sh-NC. **(D)** Cellular viability by CCK8 assay. **(E)** Quantification of invasion and migration assays. **(F)** Transwell invasion and migration assays. **(G)** Quantification of colony number in clonogenic assay. **(H)** Clonogenic assays. “ns” means “not significant”, *** means P < 0.001.

The authors apologize for this error and state that this does not change the scientific conclusions of the article in any way. The original article has been updated.

## Publisher’s note

All claims expressed in this article are solely those of the authors and do not necessarily represent those of their affiliated organizations, or those of the publisher, the editors and the reviewers. Any product that may be evaluated in this article, or claim that may be made by its manufacturer, is not guaranteed or endorsed by the publisher.

