# Supplementary figures and images for: RAB42 Promotes Glioma Pathogenesis via the VEGF Signaling Pathway
Source: Front Oncol. 2021 Nov 29;11:657029. doi: 10.3389/fonc.2021.657029 (PMC8666624; doi:10.3389/fonc.2021.657029)

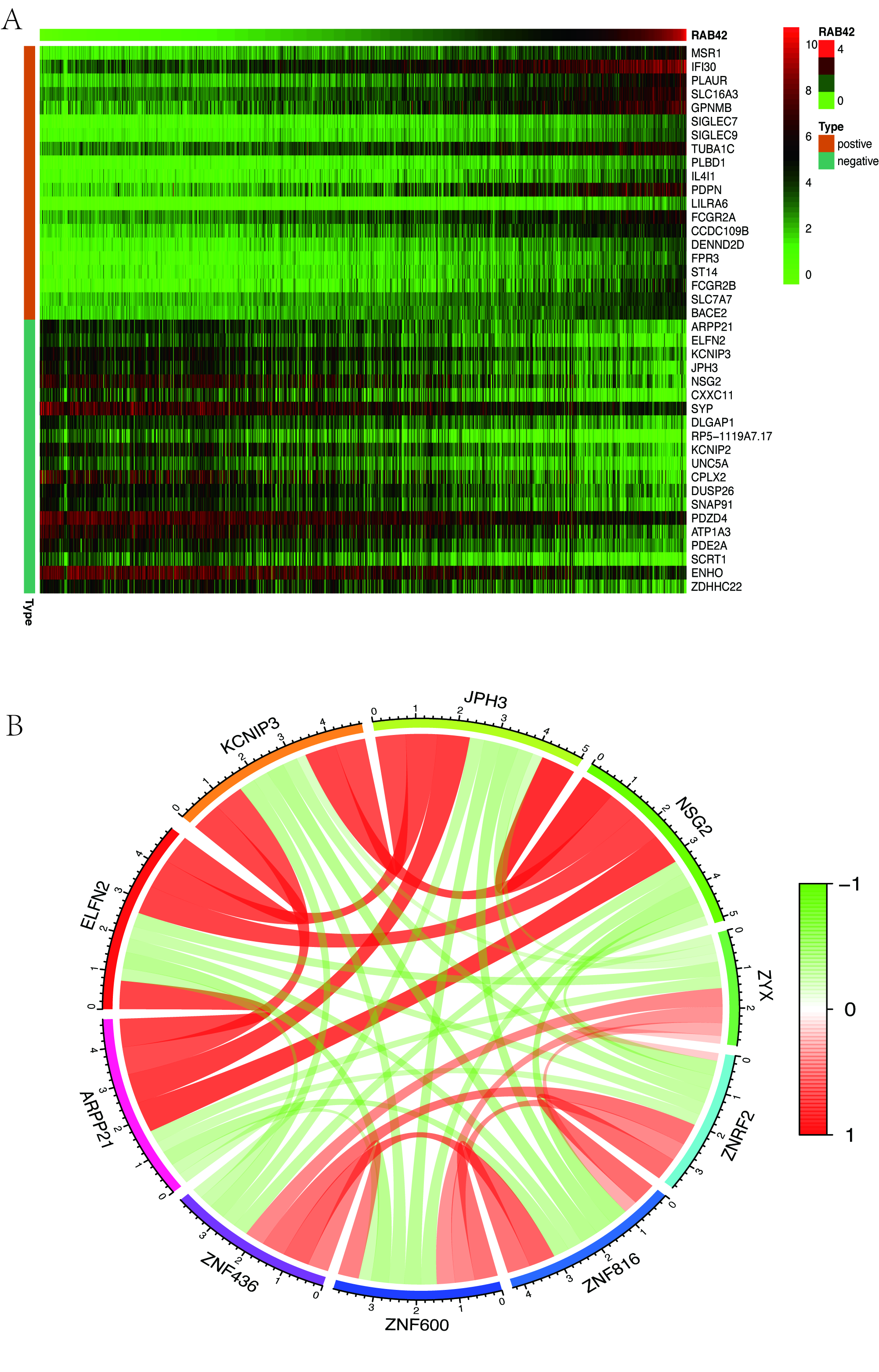

Supplement: Supplementary Figure 1 — Identification of RAB 42 Co-expression Genes. (A) Heatmap of the top twenty genes positively and negatively correlated to RAB42. (B) A circular plot of the top five genes positively and negatively associated with RAB42. Red indicates a positive correlation whereas green indicates a negative correlation. [file Image_1.tif]

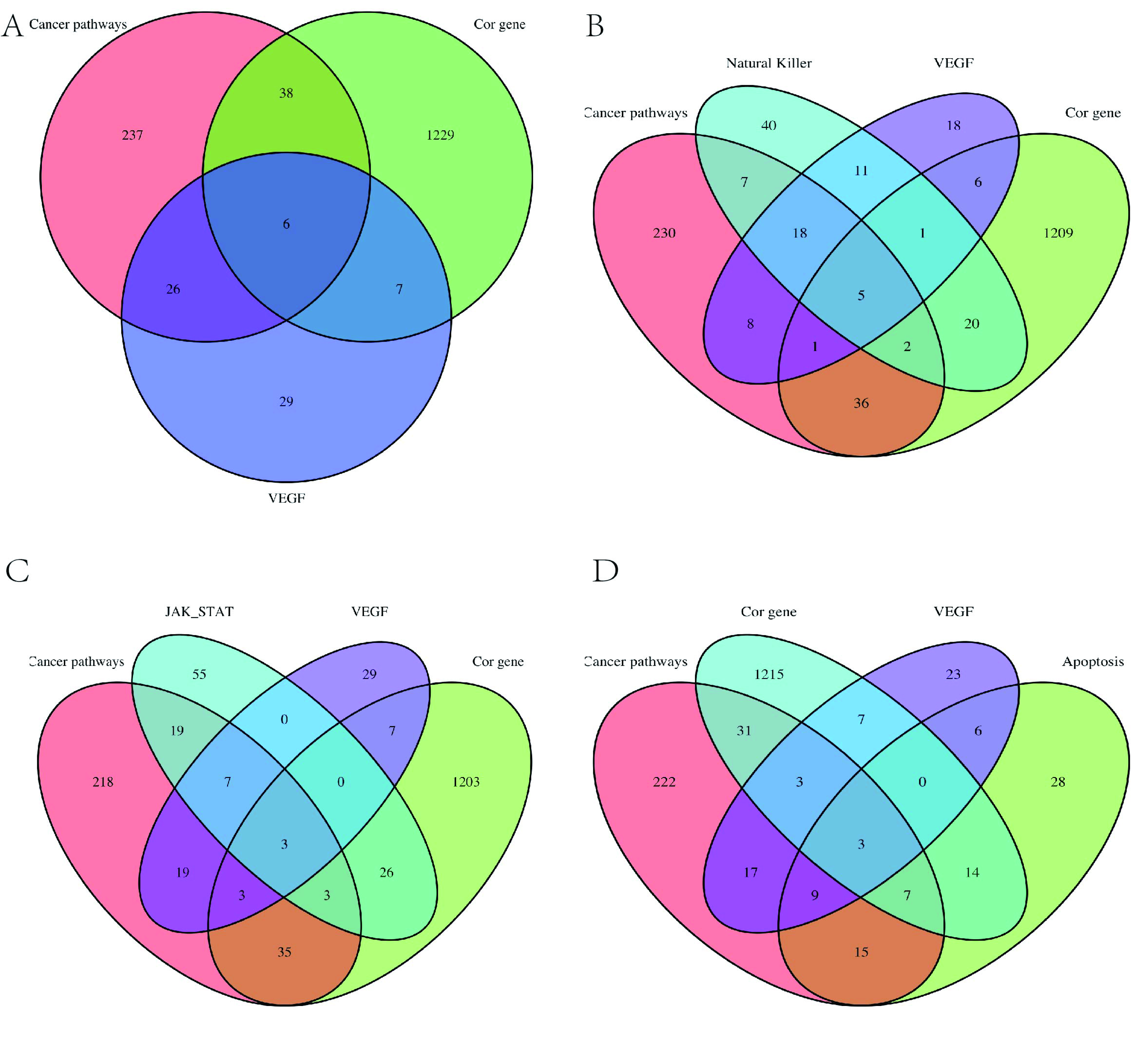

Supplement: Supplementary Figure 2 — The Venn diagrams showed the common key genes of RAB42 related genes and genes in pathways in cancer, VEGF signaling pathway, apoptosis, natural killer cell mediated cytotoxicity and JAK-STAT signaling pathways. [file Image_2.tif]

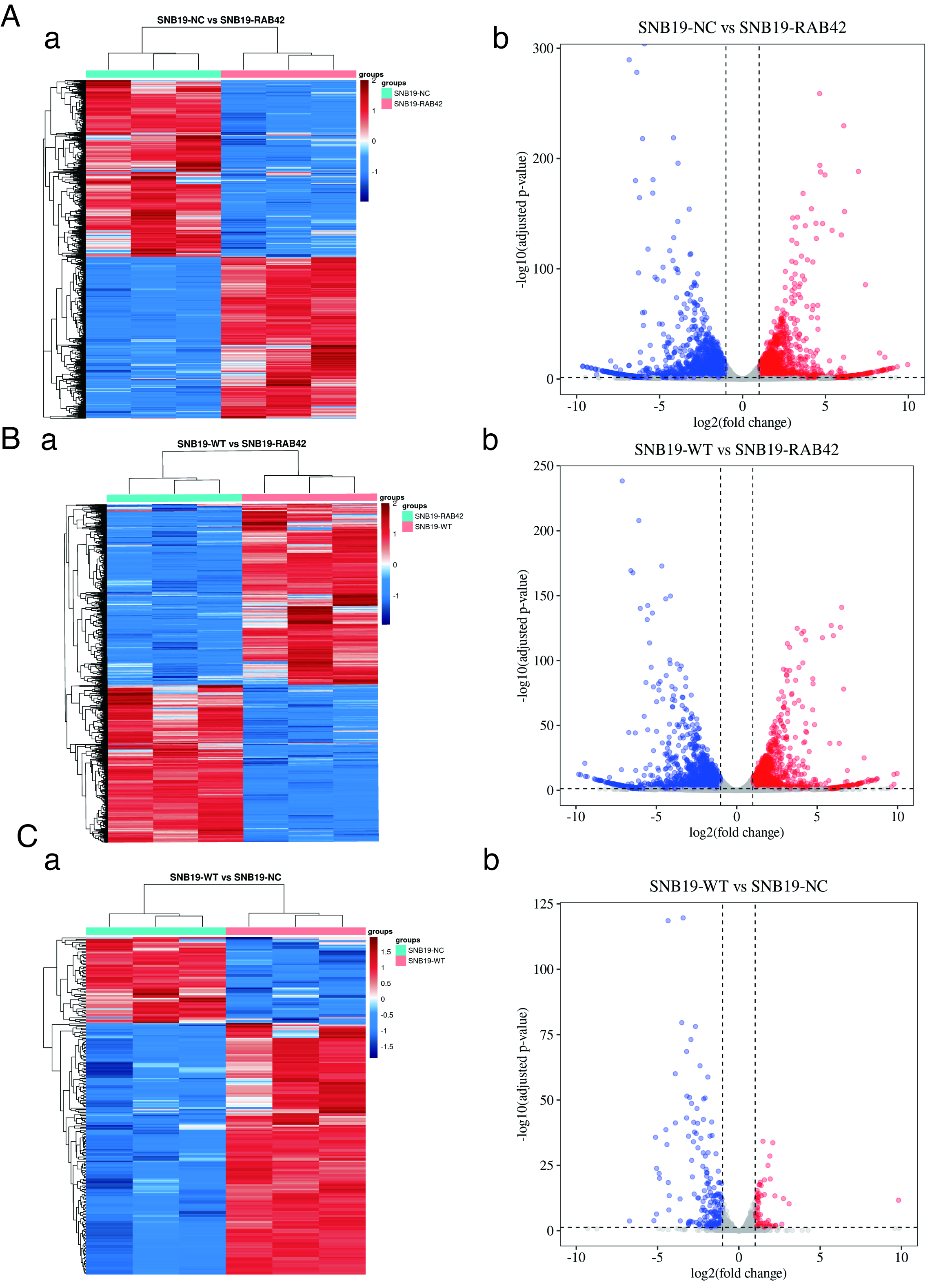

Supplement: Supplementary Figure 3 — Heatmap and Volcano Plot of the differential gene expression. [file Image_3.tif]
